# Fluorescence and electron transfer of *Limnospira indica* functionalized biophotoelectrodes

**DOI:** 10.1007/s11120-024-01114-5

**Published:** 2024-08-21

**Authors:** Nikolay Ryzhkov, Nora Colson, Essraa Ahmed, Paulius Pobedinskas, Ken Haenen, Paul J. Janssen, Artur Braun

**Affiliations:** 1 Empa. Swiss Federal Laboratories for Materials Science and Technology, Laboratory for High Performance Ceramics, 8600 Dübendorf, Switzerland; 2 Institute for Materials Research (IMO), Hasselt University, Wetenschapspark 1, 3590 Diepenbeek, Belgium; 3 IMOMEC, IMEC vzw, Wetenschapspark 1, 3590 Diepenbeek, Belgium; 4 Belgian Nuclear Research Centre, Institute for Nuclear Medical Applications, 2400 Mol, Belgium

**Keywords:** PAM-fluorescence, *Limnospira indica*, Photoelectrochemistry, Photoelectrochemical cell

## Abstract

Cyanobacteria play a crucial role in global carbon and nitrogen cycles through photosynthesis, making them valuable subjects for understanding the factors influencing their light utilization efficiency. Photosynthetic microorganisms offer a promising avenue for sustainable energy conversion in the field of photovoltaics. It was demonstrated before that application of an external electric field to the microbial biofilm or cell improves electron transfer kinetics and, consequently, efficiency of power generation. We have integrated live cyanobacterial cultures into photovoltaic devices by embedding *Limnospira indica* PCC 8005 cyanobacteria in agar and PEDOT:PSS matrices on the surface of boron-doped diamond electrodes. We have subjected them to varying external polarizations while simultaneously measuring current response and photosynthetic performance. For the latter, we employed Pulse-Amplitude-Modulation (PAM) fluorometry as a non-invasive and real-time monitoring tool. Our study demonstrates an improved light utilization efficiency for *L. indica* PCC 8005 when immobilized in a conductive matrix, particularly so for low-intensity light. Simultaneously, the impact of electrical polarization as an environmental factor influencing the photosynthetic apparatus diminishes as matrix conductivity increases. This results in only a slight decrease in light utilization efficiency for the illuminated sample compared to the dark-adapted state.

**Supplementary Information:**

The online version contains supplementary material available at 10.1007/s11120-024-01114-5.

## Introduction

The primary focus of bioelectrochemistry research applied to the design of biophotoelectrochemical cells is to achieve a maximum conversion efficiency and obtain the highest current outputs and high power density by enhancing charge transfer efficiency (Bombelli et al. 2015; Liu and Choi 2021; Saar et al. 2018; Zhu et al. 2023). However, pursuing the highest efficiency may not necessarily be the best choice for long-term autonomous operation, considering the influence of hyperosmotic or salinity stress, intense illumination, oxidative stress, and low pH on cellular processes vital for cyanobacteria (Allakhverdiev and Murata 2008; Mironov et al. 2019; Nishiyama et al. 2005; Singh et al. 2010).

The production of photocurrent in a biophotoelectrochemical cell (BPEC) can be influenced by an external applied electric bias. Careful optimization of such external electric stimulus may enhance the BPEC’s overall performance. In the case of photoelectrodes utilizing extracted photosystem I, a membrane protein complex facilitating electron transfer in light-dependent reactions across the thylakoid membrane, potentials lower than the open-circuit potential (OCP) result in an increase in cathodic photocurrent. Specifically, within the potential range of approximately + 200 to + 400 mV vs. OCP, the photocurrent remains close to zero, while higher potentials yield anodic photocurrent (Szewczyk et al. 2020). Thylakoid-based devices exhibit positive polarization leading to anodic photocurrents (Kim et al. 2016), reaching saturation at + 0.5 V vs. Ag/AgCl (Pinhassi et al. 2016). However, current and voltage are quantities composed from various contributions, derived from bioorganic and inorganic electrochemical processes. Consequently, electric signal information often lacks specificity in characterizing the influence of electric bias in complex systems.

Electrical polarization can influence photosynthesis in cyanobacteria by affecting the electron transport chain, a crucial component of the photosynthetic process (Rochaix 2011). Electrons may deviate from their predetermined, functional path inside the photosynthetic machinery, disrupting the standard electron transport chain under polarization. This alteration can also impact the energy levels within cyanobacterial cells (Muth-Pawlak et al. 2024; Szent-Györgyi 1941) or the availability of charge carriers, potentially disrupting electron flow (Szechynska-Hebda et al. 2017). Changes in pH and ion concentrations within cyanobacterial cells, induced by the change in polarization can further influence the function of various enzymes and proteins involved in light harvesting and photosynthesis (Ballottari et al. 2016; Navakoudis et al. 2023). It was demonstrated before that the thylakoid membrane polarization state, governed by an external electric field, determines charge stabilization and recombination properties of photosynthetic units (Borisevich et al. 1978; Davis et al. 2017; Knox et al. 1984).

Another factor limiting photosynthetic efficiency is the saturation of the photosynthetic apparatus, often due to the reduction of electron carriers like plastoquinone (Havaux 2020). Consequently, an external polarization bias, leading to oxidation or reduction processes, can impact the overall photosynthetic efficiency. Furthermore, chemical intermediates in the reduced state during electron transfer can result in the generation of harmful reactive oxygen species (Khorobrykh et al. 2020). Non-photochemical quenching (NPQ) mechanisms, which can safely dissipate excess light energy as heat prevent the accumulation of these reactive intermediates (Demmig-Adams and Adams 1992; Muller et al. 2001). Hence, electrical polarization can also influence NPQ.

Cyanobacteria have developed internal control mechanisms for maintaining an intracellular redox balance (Trachootham et al. 2008). Introducing a fresh electron sink compels cells to establish a new equilibrium among all electron transport routes (Rochaix 2011). Achieving a favorable equilibrium for electron transfer, without compromising long-term fitness and photosynthetic activity, is challenging without a good knowledge of the physiological state of the microorganisms in the BPECs.

The measurement of chlorophyll fluorescence is an elegant tool for probing the impact of environmental factors on photosynthesis (Kalaji et al. 2016; Schreiber 2004; Swoczyna et al. 2022). These measurements can be performed non-invasively and multiple quantitative photosynthetic parameters can be simultaneously measured. This method is applicable to plants (Lichtenthaler et al. 2005) and also for cyanobacteria (Ogawa et al. 2017). Additionally, chlorophyll fluorescence measurements can be combined with other analytical tools. In this work, chlorophyll fluorescence measurements were conducted simultaneously with current measurements under a constant applied potential bias. Chlorophyll fluorescence measurements provide valuable insights into the efficiency of photosynthetic electron transport, the functionality of photosystem II (PSII) reaction centers, and the health and stress adaptation of the photosynthetic organism while the applied external electrical field can be considered as environmental stress.

Early combinations of Pulse-Amplitude-Modulation (PAM) fluorescence spectroscopy and electrochemistry demonstrated real-time correlation between the dynamic electrochemical and fluorescence data for *Chlamydomonas reinhardtii* suspensions (Beauzamy et al. 2020, 2023). Potential correlation between the electrical current produced by the electrochemical device and certain biochemical redox processes occurring within the cells of the biofilm was demonstrated by combination of PAM and electrochemical measurements for *Chlorella* and *Synechococcus* biofilms (Ciniciato et al. 2016). Enhanced withdrawal of current from the biofilm led to heightened fluorescence at 633 nm (measured using confocal microscope), suggesting enhanced activity of the photoreceptors and thereby improved photosynthetic performance of the biofilm (Inglesby et al. 2013).

*Limnospira indica* PCC 8005 emerges as a favorable organism for the development of biophotoelectrochemical cells (BPECs). This multicellular filamentous cyanobacterium has exhibited resistance to a diversity of harsh conditions, including elevated levels of γ-rays (Badri et al. 2015a, 2015b; Yadav et al. 2021). This radiation robustness renders it as a prime candidate for utilization in space applications (Fahrion et al. 2021; Poughon et al. 2020). Opting for intact, live cyanobacteria is more favorable than utilizing inanimate cellular components since living cells have the benefit of evolutionarily optimized light harvesting for improved energy absorption and transfer, and possess the ability to adapt to stress, facilitating prolonged and autonomous operation (Tschörtner et al. 2019).

In addition to the photosynthetic components (biological cells), another essential element in BPECs is the current-collecting material. This material must satisfy various criteria, including biocompatibility, good electric conductivity, suitability for cell adhesion, and ideally, cost-effectiveness. Numerous carbon allotropes, such as diamond and diamond-like carbon (DLC), are considered good candidates for this purpose. Among these, boron-doped diamond (BDD) has attracted great interest. Boron doping distinctly improves the electronic conductivity of diamond (Ullah et al. 2015), rendering BDD electrodes very good current collectors with a broad electrochemical potential window, low background current, and remarkable chemical inertness (Gupta et al. 2009; Macpherson 2015, 2016; Panizza and Cerisola 2005; Wallny et al. 2007).

Here we report chlorophyll fluorescence measurements conducted on polarized biophotoelectrodes containing immobilized *L. indica* cyanobacteria for light-driven water splitting, cast on boron-doped diamond (BDD) as current collecting material, to estimate the effect of electrical bias as an environmental stress on the biophotoelectrochemical cell performance.

## Materials and methods

### Diamond electrode preparation

Boron-doped diamond (BDD) 180 nm thick films were synthesized on 40 × 10 mm fused silica substrates. Prior to the thin film deposition, the substrates were cleaned using an O_2_ gas discharge plasma following the method outlined before (Pobedinskas et al. 2013), in which the samples were biased negatively at 424 V in 30 standard cubic cm per minute (sccm) O_2_ flow at 5 mTorr pressure for 3 min. Diamond growth on non-diamond substrates requires artificial formation of diamond nucleation sites, therefore, substrates were seeded with diamond nanoparticles by drop-casting a water-based colloidal suspension of ultra-dispersed detonation nano-diamond of size 7 nm (0.267 g/L, NanoCarbon Institute Co., Ltd) followed by spinning the sample at 4000 revolutions per min (rpm) while flushing with deionized water for 15 s followed by spin drying.

Diamond growth was carried out in an ASTeX 6500 series microwave plasma enhanced chemical vapor deposition (MWPECVD) reactor. The BDD film growth process was performed in CH_4_/H_2_/B(CH_3_)_3_ (trimethylboron, TMB) plasma, with corresponding gas flows of 5/395/100 sccm, resulting in a methane concentration of 1% and B/C ratio of 20000 ppm (TMB gas is diluted to 1000 ppm in H_2_). The microwave power and pressure were set to 4000 W and 40 Torr, respectively. The substrate temperature of 730 °C was monitored using a Cyclops Model 52 optical infrared pyrometer (Minolta/Land) using a surface emissivity coefficient (ε) of 0.6.

### Cyanobacteria cultivation

Helical P6 morphotype cultures of axenic *Limnospira indica* PCC 8005, obtained from SCK CEN (Mol, Belgium), were cultivated in 200 mL of Zarrouk’s medium (Zarrouk 1966) with a composition optimized according to Ref. (Cogne et al. 2003). The cultivation was carried out in 550 mL cell culture flasks (CELLSTAR®, Greiner Bio-One, Vilvoorde, Belgium) under constant conditions at a temperature of 30 °C. The flasks were placed in an orbital shaker-incubator ES20/60 (Biosan, Riga, Latvia) operating at 100 rpm and 0.5 mW/cm^2^ white light illumination generated by an LED strip. Approximately 24 mL aliquots were taken from the liquid culture and distributed into small 2 mL centrifuge tubes. Cyanobacteria were harvested through a 1 min centrifugation step at 9000 rpm.

### Cyanobacteria viability test

Embedding cyanobacteria in a polymeric matrix may be a stress factor, particularly when using melted agar for gelation. Hence we studied *L. indica*, after contact with polymer: 200 μL of cell pre-culture concentrated by centrifugation were mixed with 200 μL of melted agar, 200 μL PEDOT:PSS (1:1 v/v), or 200 μL of fresh Zarrouk’s medium as control experiment. The resulting mixtures of 400 μL were used to inoculate 20 mL of fresh Zarrouk’s medium for renewed cultivation before agar and PEDOT:PSS solidified or dried. Cell growth was monitored daily by measuring absorption of cyanobacteria suspension at 440, 555, 635 and 687 nm corresponding to chlorophyll, phycoerythrocyanin, phycocyanin and again chlorophyll respectively using a SEC2000 UV-vis spectrometer (ALS Electrochemistry & Spectro-electrochemistry, ALS Co., Ltd, Tokyo, Japan). Optical micrographs were taken using a Motic AE2000 microscope with 20x objective.

### Biophotoelectrode assembly

Harvested *L. indica* PCC 8005, helical P6 morphotype, were resuspended at 1:1 v/v in 0.75% agar (Agar-agar obtained from *Rhodophyceae*, powder, CAS 9002-18-0 Sigma Aldrich, melted, 50 °C) or 0.5–1% PEDOT:PSS in water (Sigma Aldrich). The agar employed in this study contained, when specified, 100 mM [Fe(CN)_6_]^4−^ (e.g 0.75% agar containing 100 mM [Fe(CN)_6_]^4−^). For BDD samples subjected to electrochemical testing, the specimen surfaces were constrained to approximately 1 cm^2^ by sealing off the remaining area with lacomit varnish (Agar Scientific, Stansted, UK). Ten μL of bacterial cell suspensions in agar or PEDOT:PSS were drop casted onto the BDD electrode surfaces and left to solidify during 10 min in case of agar or dried on room temperature during 2 h in case of PEDOT:PSS. The resulting biophotoelectrodes are referred to as BDD/agar+P6 and BDD/PEDOT:PSS+P6 in the text. Biophotoelectrochemical cells (BPECs) consisted of a bio-anode, a platinum counter electrode, and an Ag/AgCl reference electrode.

### Measurements of variable fluorescence vs. electrical polarization

Variable chlorophyll *a* (Chl* a*) fluorescence was measured using a MICROFIBER-PAM fluorometer (Heinz Walz GmbH, Effeltrich, Germany) with a 100 µm optical fiber for fluorescence excitation and detection. This setup allowed for probing small spots of heterogeneous photosynthetic surfaces, such as microbial mats. Chl* a* fluorescence induced by 630 nm excitation was detected only at wavelengths longer than 710 nm. Excitation fluorometry pulses of 0.8 s were applied.

BDD electrodes with live cyanobacteria on top were attached to the bottom of a petri dish. Using metal wires, BDD/cyanobacteria biophotoelectrodes were connected to a Gamry Interface 1010E potentiostat (Warminster, USA) in a 3-electrode setup with a platinum counter electrode and an Ag/AgCl reference electrode in phosphate-buffered saline (PBS). Simultaneously with the Pulse-Amplitude-Modulation (PAM) fluorometry measurement, biophotoelectrodes containing viable cyanobacteria were exposed to external potential biases of 0 V, + 0.6 V, and − 0.6 V. Additionally, all samples were assessed at open circuit potential, denoted hereafter as samples without polarization, where no current is generated. Samples were dark-adapted for 20 min prior to measurement (with absence of any ambient light) to ensure the open state of all PSII reaction centers.

From the analysis of PAM variable fluorescence curves, intrinsic fluorescence of the dark-adapted sample representing the minimal fluorescence level when all PSII reaction centers are open (F_0_), and light-adapted sample under actinic illumination (630 nm, 1.6 × 10^−8^ W) (F_0_′), maximal fluorescence for the dark-adapted sample (F_m_), and maximal fluorescence for the light-adapted sample (F_m_′) were derived. To obtain F_0_ and F_m_, the dark-adapted sample was exposed to a saturation pulse closing all PSII reaction centers temporarily. After each saturating pulse, the fluorescence signal rapidly rose to a peak level F_m_. Further actinic illumination was switched on with a 40 s delay after the first saturating pulse to obtain 12 values of F_0_′ and F_m_′ by exposing the sample to 12 sequential saturating pulses with a 20 s interval. Hereby, each measurement lasted for 300 s including a dark signal measurement following the last saturating pulse. For measurements with an electrical potential bias, polarization was applied 1–3 min before the start of the PAM measurement to reach sufficient dark current stabilization. The effective quantum yield of PSII photochemistry indicating the energetic efficiency of photoautotrophy for the light-adapted state can be calculated as F_v_′/F_m_′ and a maximum quantum yield as F_v_/F_m_ for the dark-adapted state (Schreiber 2004). Non-photochemical quenching (NPQ) were calculated as (F_m_ − F_m_′)/F_m_, for each saturating pulse. The electric bias was maintained during each PAM measurement using a potentiostat in a chronoamperometry regime with the biophotoelectrode as the working electrode. Values measured with PAM fluorometer and parameters calculated from PAM data are summarized in Table 1.

**Table 1:** PAM chlorophyll fluorescence parameters and formulas

Parameter	Description	Formula
F_0_	Minimal fluorescence yield in the dark-adapted state	Measured directly when the sample is in the dark-adapted state and no actinic light is present
F_m_	Maximal fluorescence yield in the dark-adapted state	Measured directly after applying a saturating pulse of light to the dark-adapted sample
F_v_	Variable fluorescence in the dark-adapted state	F_v_ = F_m_ − F_0_
F_0_′	Minimal fluorescence yield in the light-adapted state	Measured directly when the sample is in the light-adapted state
F_m_′	Maximal fluorescence yield in the light-adapted state	Measured directly after applying a saturating pulse of light to the light-adapted sample
F_v_′	Variable fluorescence in the light-adapted state	F_v_′ = F_m_′ − F_0_′
F_v_′ /F_m_′ or Φ_PSII_	Effective quantum yield of PSII in the light-adapted state/ Quantum efficiency of PSII photochemistry	F_v_′ /F_m_′ = Φ_PSII_= (F_m_′ − F_0_′)/F_m_′
F_v_/F_m_	Maximal quantum yield of PSII in the dark-adapted state	F_v_/F_m_ = (F_m_ − F_0_)/ F_m_
NPQ	Non-photochemical quenching parameter	NPQ = (F_m_ − F_m_′)/F_m_′
ETR	Electron Transfer Rate	ETR = ΔF/F_m_′ × PAR × 0.5 × 0.84 where PAR is the photosynthetically active radiation
Alpha	Initial slope of the light response curve	Determined from ETR vs PAR curve

## Results and discussion

### Biophotoelectrodes assembly

**Fig. 1:**
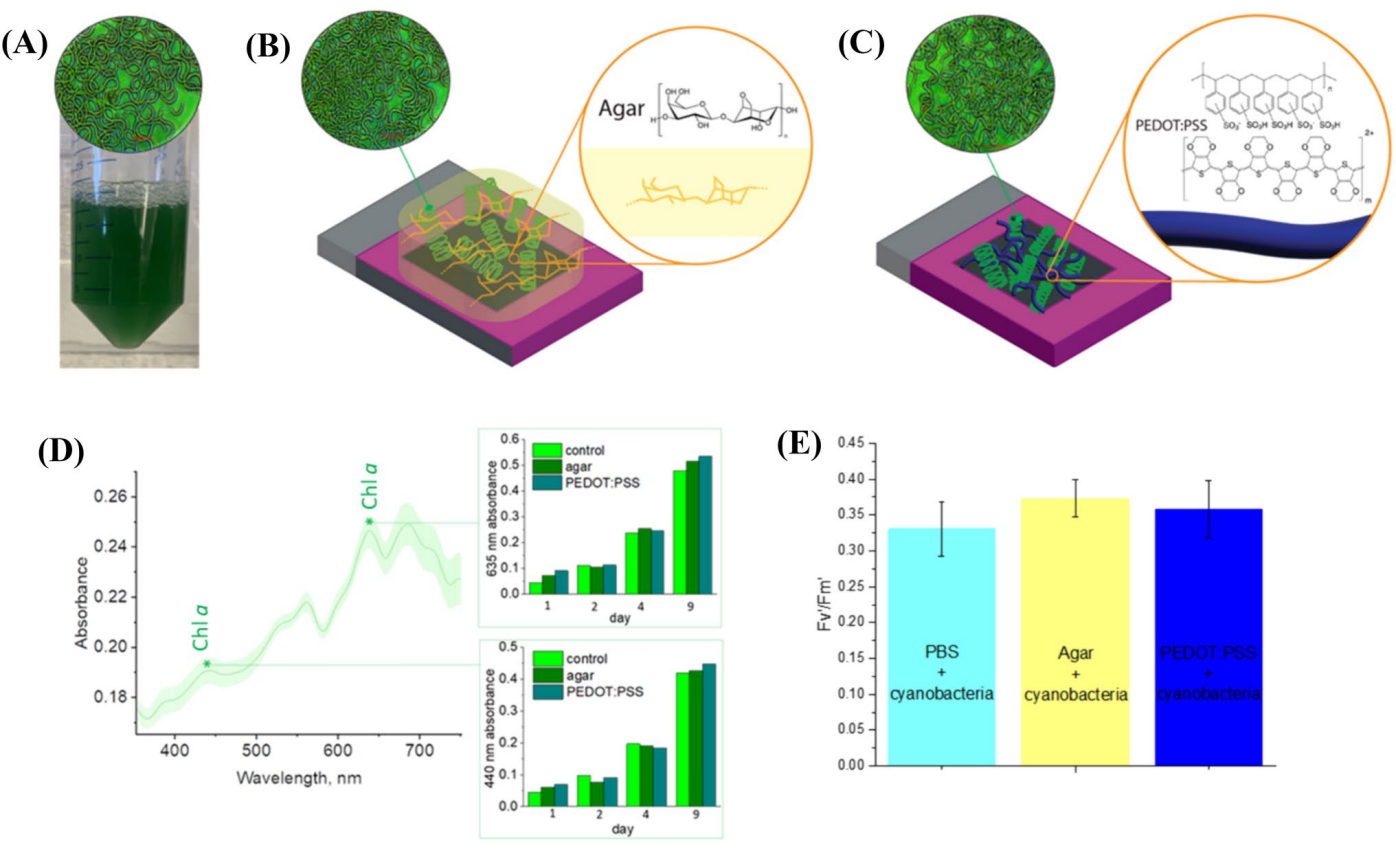
**A** *L. indica* cyanobacteria in Zarrouk medium suspension obtained from pure pre-culture (optical microscopy imaging, 40×). **B** *L. indica* cyanobacteria embedded in agar hydrogel (optical microscopy imaging, 40×). **C** *L. indica* cyanobacteria embedded in PEDOT:PSS (optical microscopy imaging, 40×). **D** Average absorbance spectra of *L. indica* in Zarrouk medium suspension obtained from pure pre-culture, pre-culture mixed with hot agar and pre-culture mixed with PEDOT:PSS on the 4th day of cultivation, green shading is a standard deviation, bar charts on the right demonstrate time evolution of main Chl *a* absorbance bands. **E** Average efficient photochemical yield of cyanobacteria in free suspension and embedded in agar or PEDOT:PSS

Cyanobacteria (Fig. 1A) were applied to the surface of the BDD electrode using two immobilization strategies: (i) agar hydrogel and (ii) PEDOT:PSS layer (Fig. 1B, C). The choice for agar is based on its capability to form robust hydrogels that firmly adhere to the surface; whereas PEDOT:PSS is chosen for its characteristics as a translucent conductive polymer (Jing et al. 2023; Wolfe et al. 2021). The procedure for cyanobacterial immobilization involves mixing a concentrated cell suspension with melted agar (above its melting point, around 50 °C).

As mixing *L. indica* cyanobacteria with either melted agar or PEDOT:PSS solution may be a stressful event possibly leading to cellular damage, we conducted an examination of the viability of cyanobacteria following their interaction with these polymers, which are in fact intended to have not only an immobilizing but also a stabilizing, protective function. To assess this, cyanobacteria suspended in agar or PEDOT:PSS were used to reinoculate Zarrouk’s medium and subsequently cultivated under standard conditions (see Methods). Daily aliquots of these solutions were collected for light absorption measurements.

The general trend of absorbance increase was consistent among all cultures, indicating that the interaction with melted agar or the conductive polymer had little or no malign influence on the reproductive ability and viability of the cyanobacterial cells. Fig. 1D depicts changes in absorbance at 440 and 635 nm corresponding to chlorophyll over time for *L. indica* PCC 8005 recultured from cells immobilized in agar, PEDOT:PSS, and a liquid bacterial suspension. Since chlorophyll is reported to be a good proxy for cyanobacterial biomass [68], it can be concluded that there is no or very little difference in primary production between these cultures. Additionally, there is no significant (ANOVA, significance level 5%) difference in photosynthesis efficiency parameters, such as photosynthetic yield between suspended and PEDOT:PSS immobilized cyanobacteria as well as PEDOT:PSS and agar immobilized. However, light utilization efficiency for agar immobilized cyanobacteria is higher than for free-floating cells which might be attributed to a transient heat shock response (ANOVA, significance level 5%) (Fig. 1E). From this we can conclude that the mixing of cell suspensions with agar or PEDOT:PSS has no discernable effect on bacterial growth.

### Chlorophyll fluorescence measurements–PAM induction curves

The kinetics of PSII quantum yield (Φ_PSII_), non-photochemical quenching (NPQ), and minimum F_0_′ and maximum F_m_′ chlorophyll (Table 1) fluorescence provide essential information for analyzing the photo acclimation of the photosynthetic apparatus during a transition from darkness to light in cyanobacteria, algae, and plants. Non-destructive chlorophyll fluorescence is a valuable tool to study the photo physiology and ecology of photosynthetic organisms.

**Fig. 2:**
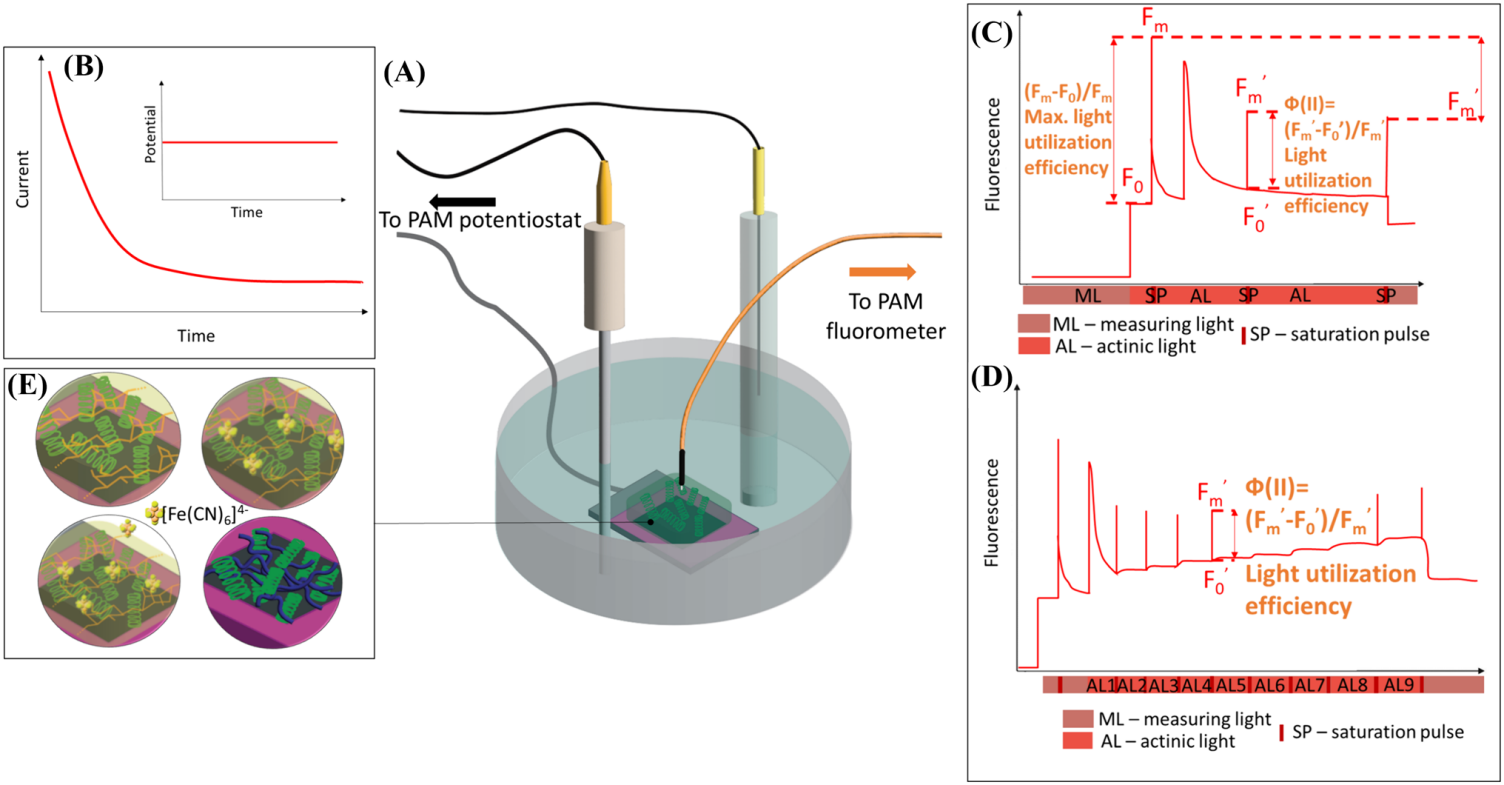
**A** Experimental setup for electrically polarized biophotoelectrode PAM measurements. **B** Schematic representation of chronoamperometric measurements. **C** Schematic representation of fluorescence quenching analysis using the saturation pulse method. **D** Schematic representation of rapid light curve and parameters of photosynthesis activity derived from it. **E** our types of biophotolelectrodes used in this research: cyanobacteria embedded in agar hydrogel in PBS solution (top left), cyanobacteria embedded in agar hydrogel containing [Fe(CN)_6_]^4−^ (top right) in PBS solution, cyanobacteria embedded in agar hydrogel containing [Fe(CN)_6_]^4−^ (top right) in PBS solution containing [Fe(CN)_6_]^4−^, **E** cyanobacteria embedded in PEDOT:PSS in PBS solution

Typically, environmental factors such as light intensity, temperature, and the chemical composition of the surrounding medium (e.g., groundwater, air) are considered when assessing the photosynthetic performance of phototrophs. For example, Menguy and co-workers used Pulse-Amplitude-Modulation (PAM) fluorometry to assess the effect of algicid compounds on algal extracts (Menguy et al. 2020). However, when live cyanobacteria are used in biophotovoltaic devices, the interruption of the photosynthetic electron generation and transfer pathways is a critical factor in device performance. To attain optimal performance in devices relying on bacteria, it is often necessary to apply external electrical bias (Pinhassi et al. 2016; Szewczyk et al. 2020). Designing self-biased cells can also be an option (Wang et al. 2013). This helps to surmount thermodynamic limitations and counterbalances energy losses arising from electrical resistance. Nevertheless, to the best of our knowledge, there has been no systematic research on the influence of electrical polarization on the functionality of the photosynthetic apparatus. To study the influence of electrical polarization required for photocurrent production by biophotoelectrodes, BDD electrodes with live cyanobacteria on top were polarized using a potentiostat in a 3-electrode setup. Simultaneously, chlorophyll fluorescence was measured using PAM fluorometry with excitation light provided and fluorescence collected using a microfiber (Fig. 2A).

Chl* a* re-emits light energy in the red band, with most of the fluorescence coming from PSII. When no photons are absorbed by cyanobacteria, their PSII reaction centers are considered open, and no electrons are donated from water splitting. This results in minimal chlorophyll fluorescence (F_0_).

Before conducting measurements, biophotoelectrodes were dark-adapted to open PSII reaction centers. Subsequently, samples were exposed to measuring light to determine the dark fluorescence of chlorophyll (F_0_) (Fig. 2B).

Oxygenic photosynthesis in cyanobacteria is initiated by light-induced charge separation in chlorophyll. An electron hole is transferred to a water oxidation catalytic center. The electron is transferred to the primary quinone electron acceptor Q_A_ (Govindjee and Björn 2017). A short pulse of high-intensity light is sufficient to reduce all of the primary electron acceptor quinone co-factor Q_A_ and close all reaction centers. When reaction centers are closed, no photosynthesis can occur, and all excess energy is dissipated as fluorescence, reaching the maximum fluorescence (F_m_) (Fig. 2B). PSII maximum quantum yield (F_v_/F_m_) can be calculated using the equation F_v_/F_m_ = (F_m_ − F_0_)/F_m_. This yield is maximal when the sample is in a dark-adapted state.

The second part of the measurement involves exposing live cyanobacteria to light, exciting pigment molecules and driving photosynthesis. Under these conditions, some reaction centers are engaged in photosynthesis, resulting in closed centers (Q_A_ reduced). The fluorescence signal F_0_′ is expected to be between F_0_ and F_m_, proportional to the fraction of closed reaction centers. F_m_′ represents the maximum fluorescence obtained by applying a saturating pulse. F_m_′ is usually lower than F_m_ due to non-photochemical quenching, a process that diverts light energy from PS II (Fig. 2B).

Effective PSII quantum yields (Φ_PSII_) can be calculated using the equation Φ_PSII_ = (F_m_′ − F_0_′)/F_m_′. However, in some cases for cyanobacteria F_m_′ may exceed F_m_, leading to negative values of non-photochemical quenching (NPQ) (Ogawa et al. 2017). NPQ mechanisms prevent over-reduction of the photosynthetic machinery by thermally dissipating excessive energy, and it can be quantified using the equation NPQ = (F_m_ − F_m_′)/F_m_ (Fig. 2B).

These measurements provide valuable insights into the photosynthetic performance and health of photosynthetic organisms by analyzing changes in the fluorescence signal during a series of light pulses. The fluorescence pattern is modulated by a weak measuring light superimposed with short saturating flashes of light, allowing the study of photosynthetic efficiency and energy flow within the photosynthetic apparatus.

Photosynthesis-irradiance curves were plotted to study the relationship between light intensity and photosynthesis under external polarization using data derived from rapid light curves (Fig. 2C). Electron transport rates through PSII (ETR(II)) at a given photosynthetically active radiation (PAR) can be calculated using the equation ETR(II) = Φ_II_ × PAR × 0.84 × 0.5. By plotting ETR against PAR, the maximum photosynthesis rate (*P*_max_) can be determined as ETR(II) measured at saturating light intensity.

### Hydrogel immobilized cyanobacteria

**Fig. 3:**
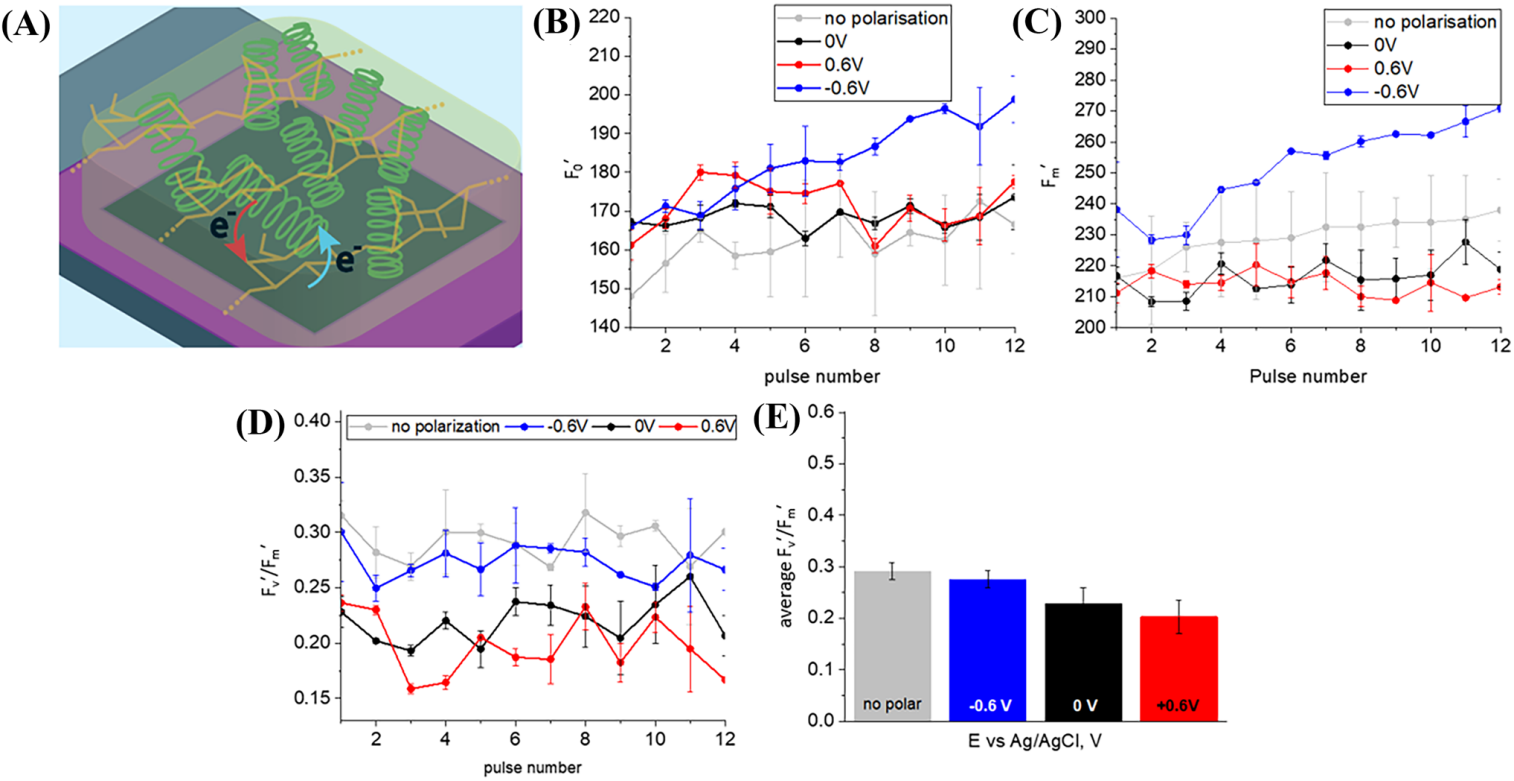
**A** Scheme of charge transfer associated with anodic (red arrow) and cathodic (blue arrow) currents in biophotoelectrode containing *L. indica* cyanobacteria embedded in mediator-free agar, **B** Data on steady-state fluorescence (F_0_′) are derived from the PAM curves. **C** Data on the maximum chlorophyll fluorescence of light-adapted biophotoelectrodes. **D** The effective quantum yield of photoconversion calculated as (F_m_′ − F_0_′)/F_m_′, and **E** The average effective quantum yield of photoconversion

Figure S1 A presents PAM variable fluorescence curves for *L. indica* immobilized in agar gel on top of a BDD electrode (Fig. 3A) with + 0.6 V, − 0.6 V and 0 V potential biases as well as without external polarization. The increase of the fluorescence intensity is characteristic for the closure of reaction centers: electron transport starts and quinone-type acceptors (Q_A_) are reduced. This process is faster than Q_A_ re-oxidation due to electron transfer to another electron acceptor on further steps of photosynthetic electron transport chain. This results in accumulation of reduced primary electron-acceptors Q_A_ and light energy dispersion by fluorescence. The fluorescence level F_0_′ increases after the onset of the actinic light and keeps a higher level than the F_0_ level in the dark, demonstrating an imbalance between photochemical and non-photochemical quenching, i.e., the increase of photochemical quenching is larger than the decrease of non-photochemical quenching (Ogawa et al. 2017). Both minimum (Fig. 3B) and maximum (Fig. 3C) fluorescence levels extracted from the PAM curve (Fig. S1A) are increasing with time when bacteria immobilized in agar hydrogel are polarized at − 0.6 V and illuminated with actinic light. This increasing trend for F_0_′ may indicate a further increase of photochemical quenching and a decrease in non-photochemical quenching under negative potential bias because more and more photocenters possibly become reduced. The growth of F_m_′, representing the maximum fluorescence under actinic light, might be related to the increase in the F_0_′ baseline since the photosynthetic yield of the photosystem (F_v_′/F_m_′) appears to remain more or less constant (as shown in Fig. 3D). Given the synchronized growth of minimal and maximum fluorescence at − 0.6 V (Fig. 3B, D), the value of F′_v_/F_m_, where F_v_′ = F_m_′ − F_0_′ is a variable fluorescence, which is proportional to the fracture of active photocenters doesn’t show the same trend and remains constant (Fig. 3D). By comparing the photosynthetic yield obtained from biophotoelectrodes operated under different polarization conditions (Fig. 3E) we observed that the photoconversion yields at 0 V and + 0.6 V were nearly the same but lower than the value obtained for the negatively polarized ( − 0.6 V) and non-polarized samples, albeit that this difference is only slightly above the threshold of statistical significance.

Also, the photoconversion yield indicating the light utilization efficiency for *L. indica* cyanobacteria, as immobilized on the biophotoelectrode, is lower than the average value of 0.4–0.6 reported elsewhere for a number of cyanobacteria in free cultures, including *Arthrospira platensis* NIES-39 and *Anabaena* sp. PCC 7120 (Misumi et al. 2016), or for the green alga *Chlorella* immobilized in alginate gel (Ng et al. 2017). Further, increase in F_m_′ rather than a decrease typical for plants was observed throughout the process of light acclimation in cyanobacteria (Ogawa et al. 2017). The apparent F_m_ value in dark-acclimated cells does not represent the actual maximum level of chlorophyll fluorescence for many cyanobacterial species.

**Fig. 4:**
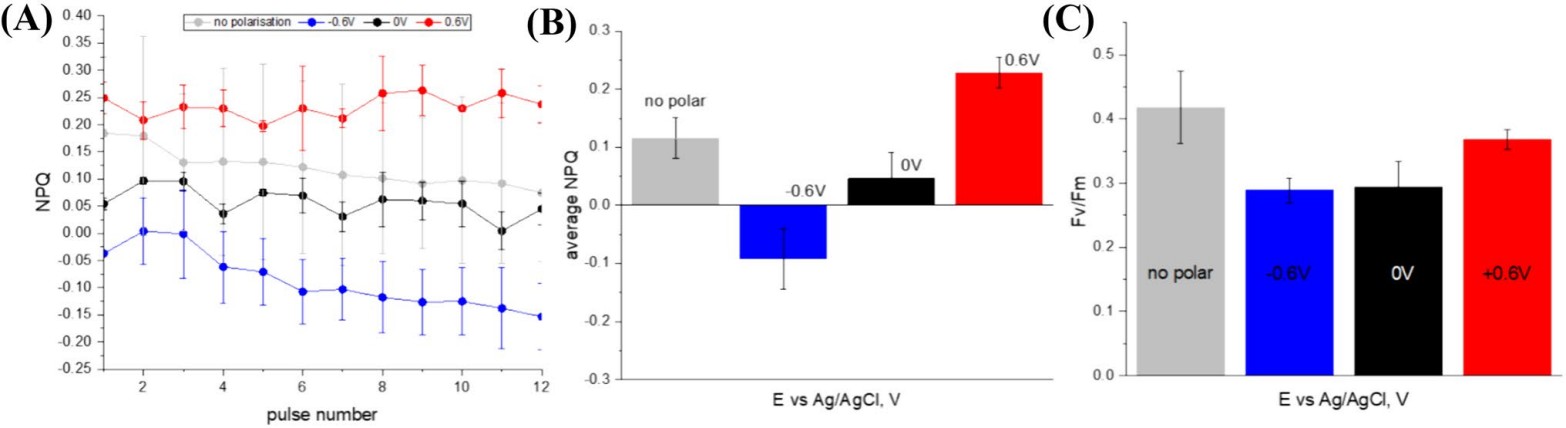
**A** NPQ values derived from PAM curves, **B** average NPQ values, **C** average effective quantum yield of photoconversion for BDD/agar+P6 in PBS solution

Since cyanobacterial respiratory electron transport uses the same PQ pool in thylakoid membranes as photosynthetic electron transport (Aoki and Katoh 1982; Ogawa and Sonoike 2015), the F_m_ level is already quenched in the dark and a determination of the maximum photoconversion yield and NPQ ((F_m_ − F﻿_m_﻿′)/F_m_) results in that case negative values. However, for *L. indica* immobilized in agar hydrogel on top of BDD such a feature’typical’ for cyanobacteria was only observed in the case of − 0.6 V polarization (Fig. 4A, B). The maximum fluorescence F_m_, which is used for the calculation of NPQ ((F_m_ − F_m_′)/F_m_′), was measured separately for each electrical polarization tested. Maximum quantum yields were determined as 0.41 ± 0.05 for non-polarized sample, 0.29 ± 0.02 for − 0.6 V, 0.29 ± 0.04 for 0 V, and 0.37 ± 0.02 for 0.6 V (Fig. 4C).

**Fig. 5:**
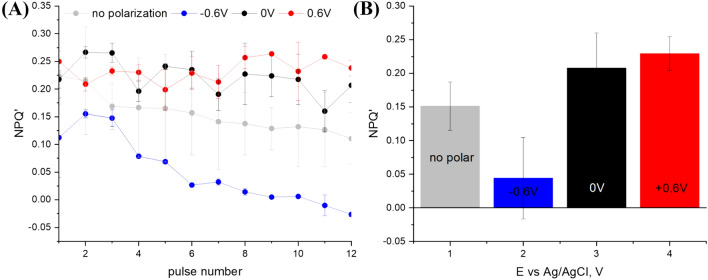
**A** NPQ values derived from PAM curves using F_m_ value measured at + 0.6 V, **B** average NPQ values for different polarizations.

Essentially, precise F_m_ and NPQ values can be assessed in cyanobacteria under conditions where PQ is oxidized, such as during blue light exposure or illumination in the presence of the PSII inhibitor 3-(3,4-dichlorophenyl)-1,1-dimethylurea (DCMU) (Ogawa et al. 2017). Since a positive potential bias can promote PQ pool oxidation, we believe F_m_ measured in these conditions can be used for NPQ estimation. NPQ values recalculated given that real maximum fluorescence value of dark adapted state can be obtained with positive potential applied ( + 0.6 V) demonstrate that NPQ mechanism is suppressed at negative potential (Fig. 5).

### Introducing electron shuttle for enhanced charge transport

Efficiency of energy harvesting from photosynthetic organisms in biophotoelectrochemical systems exploits the phenomenon of extracellular electron transport (EET). However, since cyanobacteria rely on photosynthesis as their main energy source, EET in photosynthetic organisms is, in general, limited compared to other bacteria. Photocenters are located in the thylakoid membranes that are separated by the cytoplasma and the outer membrane from the external environment. Therefore, even if cells are in close contact with the electrode, EET activity is limited (Kusama et al. 2022). Individual *L. indica* cells are packed into trichomes surrounded by an extra sheath being an additional limitation for EET. Moreover, owing to the helical shape of PCC 8005 (morphotype P6) trichomes fewer cells are in direct contact with the substrate. As agar hydrogel is not conductive itself, current enhancement is solely expected through the added mediator [Fe(CN)_6_]^4−^. When using live cyanobacteria immobilized at the electrode, there are two strategies to introduce this mediator into the system. It can be either mixed with the electrolyte or embedded in the hydrogel together with the cyanobacteria. Figure S2 demonstrates the increase of current production of BDD/agar+P6 photoelectrode in the presence of [Fe(CN)_6_]^4−^ both in agar hydrogel and in liquid electrolyte in which the photoelectrode is immersed. This increase is clearly more pronounced at positive polarization.

**Fig. 6:**
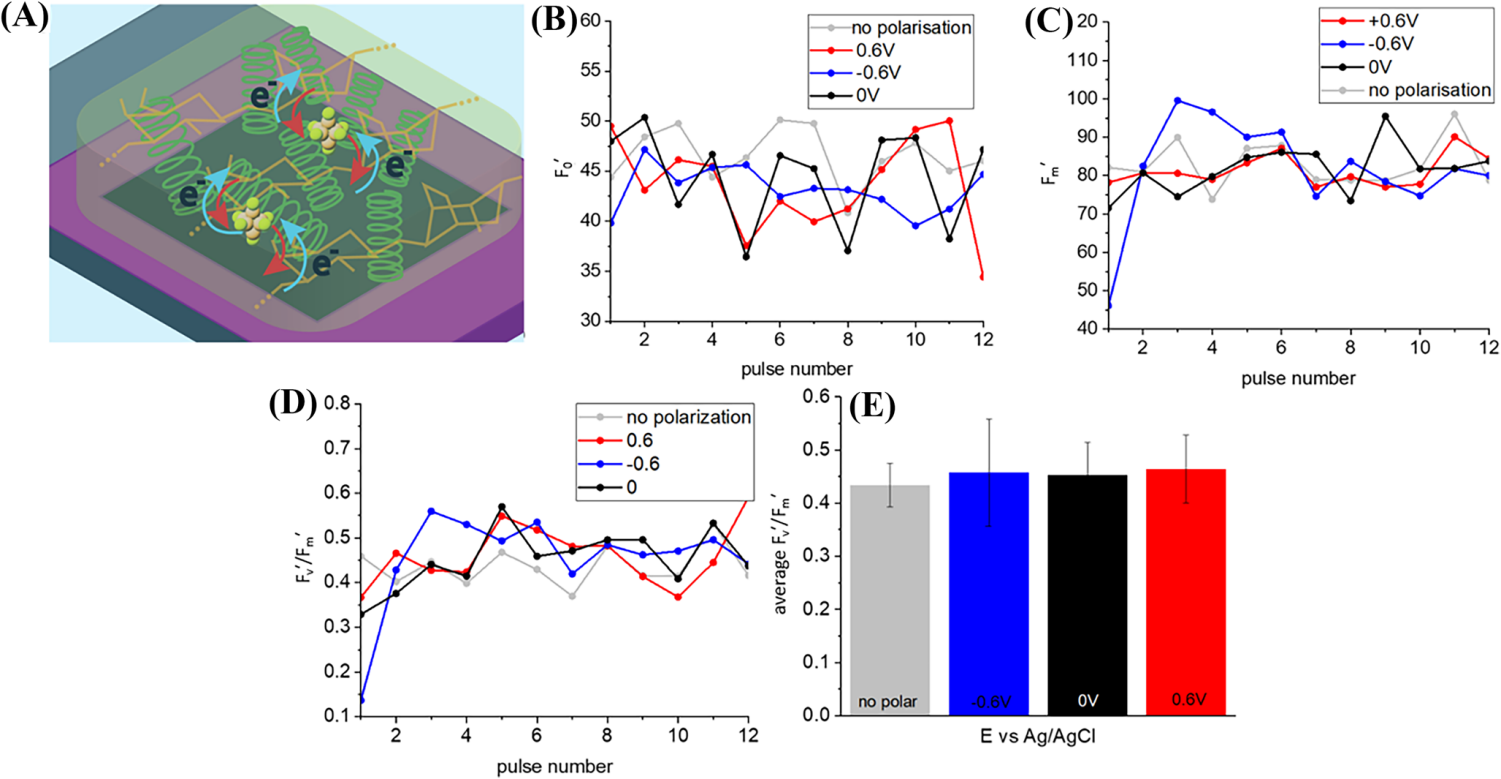
**A** Scheme of charge transfer associated with anodic (red arrow) and cathodic (blue arrow) currents in a biophotoelectrode setup consisting of cyanobacteria embedded in agar with [Fe(CN)_6_]^4−^ mediator. **B** Data on steady-state fluorescence (F_0_′) derived from the PAM curves. **C** Data on the maximum chlorophyll fluorescence of light-adapted biophotoelectrodes. **D** The effective quantum yield of photoconversion, calculated as (F_m_′ − F_0_′)/F_m_′ and **E** The average effective quantum yield of photoconversion.

Steady-state fluorescence under actinic illumination F_0_′ (Fig. 6B) derived from PAM curves (Fig. S1B) of cyanobacteria embedded in agar together with [Fe(CN)_6_]^4−^ mediator placed on top of the BDD (Fig. 6A) as well as maximum chlorophyll fluorescence of light adapted sample F_m_′ (Fig. 6C) didn’t show features of cyanobacteria embedded in mediator free agar, namely elevated values at − 0.6 V polarization and rising trend (Figs. 6B, C vs. 3B, C).

For the three potentials tested, the effective quantum yield of photoconversion is higher when compared to photosynthetic cells embedded in mediator free agar (as shown in Fig. 6D, E compared to Fig. 3D, E). The decrease in fluorescence F_0_′ and F_m_′ can be explained by the presence of electroactive [Fe(CN)_6_]^4−^ which probably facilitates electron transfer (Fig. S2) and presumably captures ’excessive charges’ before they relax, resulting in fluorescence. However, the influence of the potential bias on cyanobacteria embedded in agar containing [Fe(CN)_6_]^4−^ mediator remains unclear, as differences in photosynthetic yield between different potentials are not statistically significant (ANOVA, significance level 5%). The only noticeable feature is an extremely low F_m_′ value for -0.6V polarized sample at the beginning of light adaptation under actinic light (Fig. 6C). This indicates reduced photosynthetic activity, which may be attributed to stress because of negative reductive potential bias. F_m_′ value increases with the time but more rapidly (Fig. 6C, D) in comparison with mediator-free agar hydrogel (Fig. 3C). The maximum photoconversion yield, and consequently F_v_′/F_m_′, for the *L. indica* cyanobacteria in agar hydrogel containing [Fe(CN)_6_]^4−^ also increases rapidly and reaches value similar to non-polarized and + 0.6 V polarized biophotoelectrodes. This value stays stable during further actinic illumination. At the same time cyanobacteria in [Fe(CN)_6_]^4−^-free agar hydrogel demonstrate stable fluorescence values within the observation time since F_0_′ values of the chlorophyll fluorescence of light-adapted biophotoelectrodes in the absence of saturation pulses are also showing an rising trend.

**Fig. 7:**
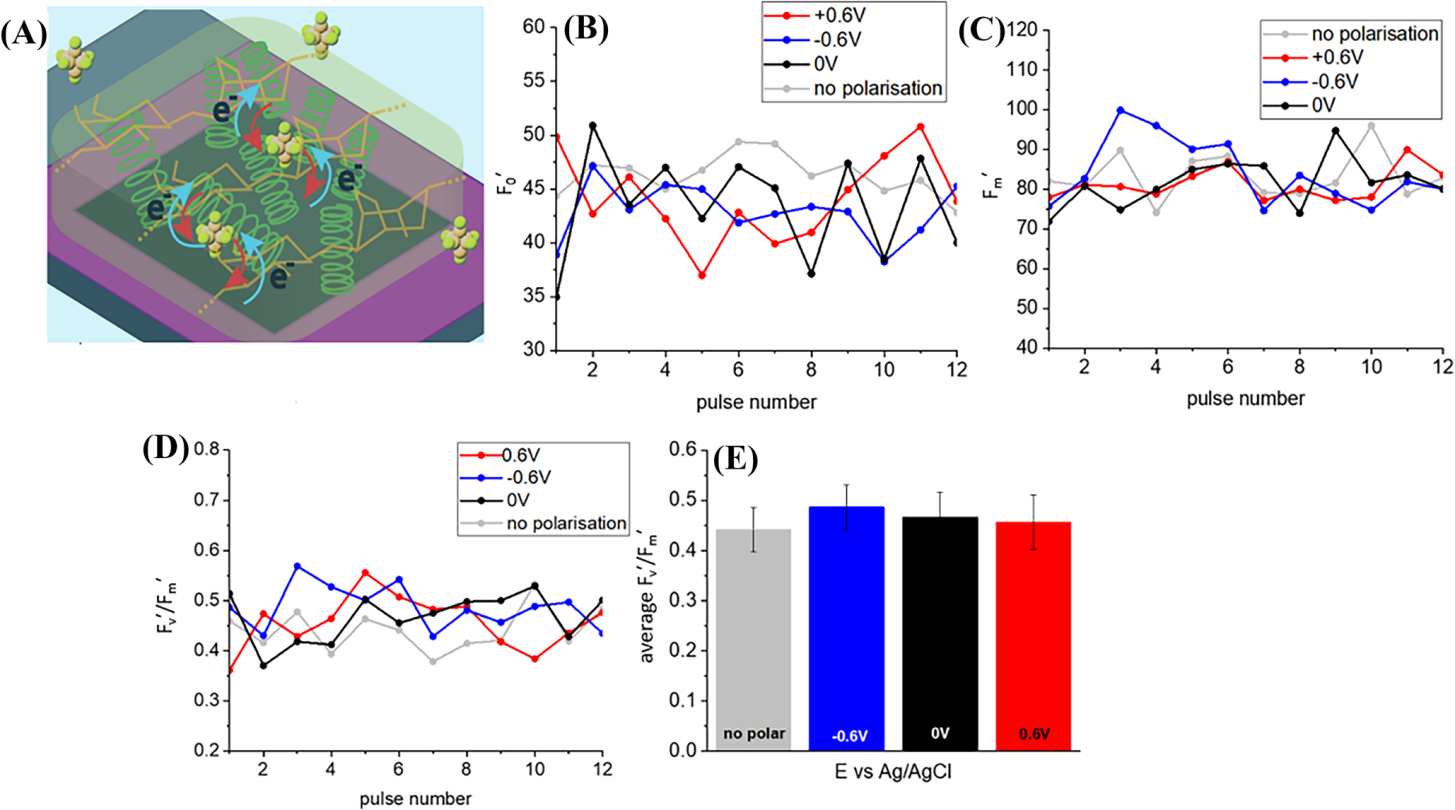
**A** Scheme of charge transfer associated with anodic (red arrow) and cathodic (blue arrow) currents in biophotoelectrode containing cyanobacteria embedded in agar containing [Fe(CN)_6_]^4−^ mediator in electrolyte containing [Fe(CN)_6_]^4−^ mediator. **B** Data on steady-state fluorescence (F_0_′) derived from PAM curves. **C** Data on the maximum chlorophyll fluorescence of light-adapted biophotoelectrodes. **D** The effective quantum yield of photoconversion calculated as (F_m_′ − F_0_′)/F_m_′, and **E** Average effective quantum yield of photoconversion at different polarizations.

When comparing results obtained from *L. indica* in agar hydrogel containing [Fe(CN)_6_]^4−^ on top of BDD in mediator-free buffer with the results of the hydrogel and electrolyte containing [Fe(CN)_6_]^4−^ (Fig. 7A–C, Fig. S1C), no significant difference (ANOVA, significance level 5%) in average effective quantum yields of photoconversion were observed (as shown in Fig. 7D, E).

Even though average values of effective quantum yield are almost identical (Figs. 6E and 7E), some differences can be observed. For instance, in Fig. 7C, there is no adaptation period with fluorescence increase at the beginning of actinic illumination.

**Fig. 8:**
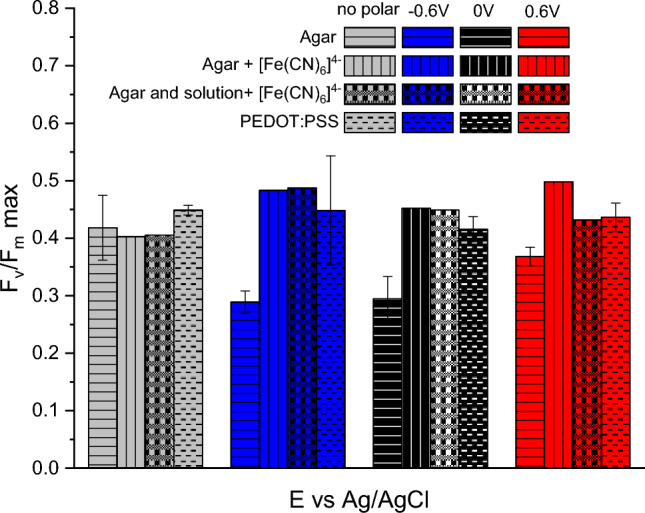
Maximum photoconversion yields for cyanobacteria embedded in mediator-free agar in PBS solution, agar with [Fe(CN)_6_]^4−^ in PBS solution and agar with [Fe(CN)_6_]^4−^ in [Fe(CN)_6_]^4−^ containing PBS solution, and in PEDOT:PSS.

Figure 8 compares maximum photoconversion yield (F_v_/F_m_) values for systems with the charge [Fe(CN)_6_]^4−^ only in agar vs. the system where [Fe(CN)_6_]^4−^ is present both in agar and in the electrolyte solution.

The results presented in Fig. 8 support the finding primarily drawn from Figs. 3, 6 and 7, in that the addition of charge carrier [Fe(CN)_6_]^4−^ to the system leads to an increase of both the maximum photoconversion yield of dark adapted cyanobacteria F_v_/F_m_ and the average effective photoconversion yield of light adapted cells Φ_PSII_. However, the maximum light utilization efficiency of non-polarized BDD/agar+P6 bioelectrode is not influenced by the presence of [Fe(CN)_6_]^4−^.

The standard deviation of average NPQ values exceeds the values themselves, making it challenging to propose any interpretation of these data (Fig. S3). As a consequence, NPQ values are not discussed further.

### Conductive polymer matrix

**Fig. 9:**
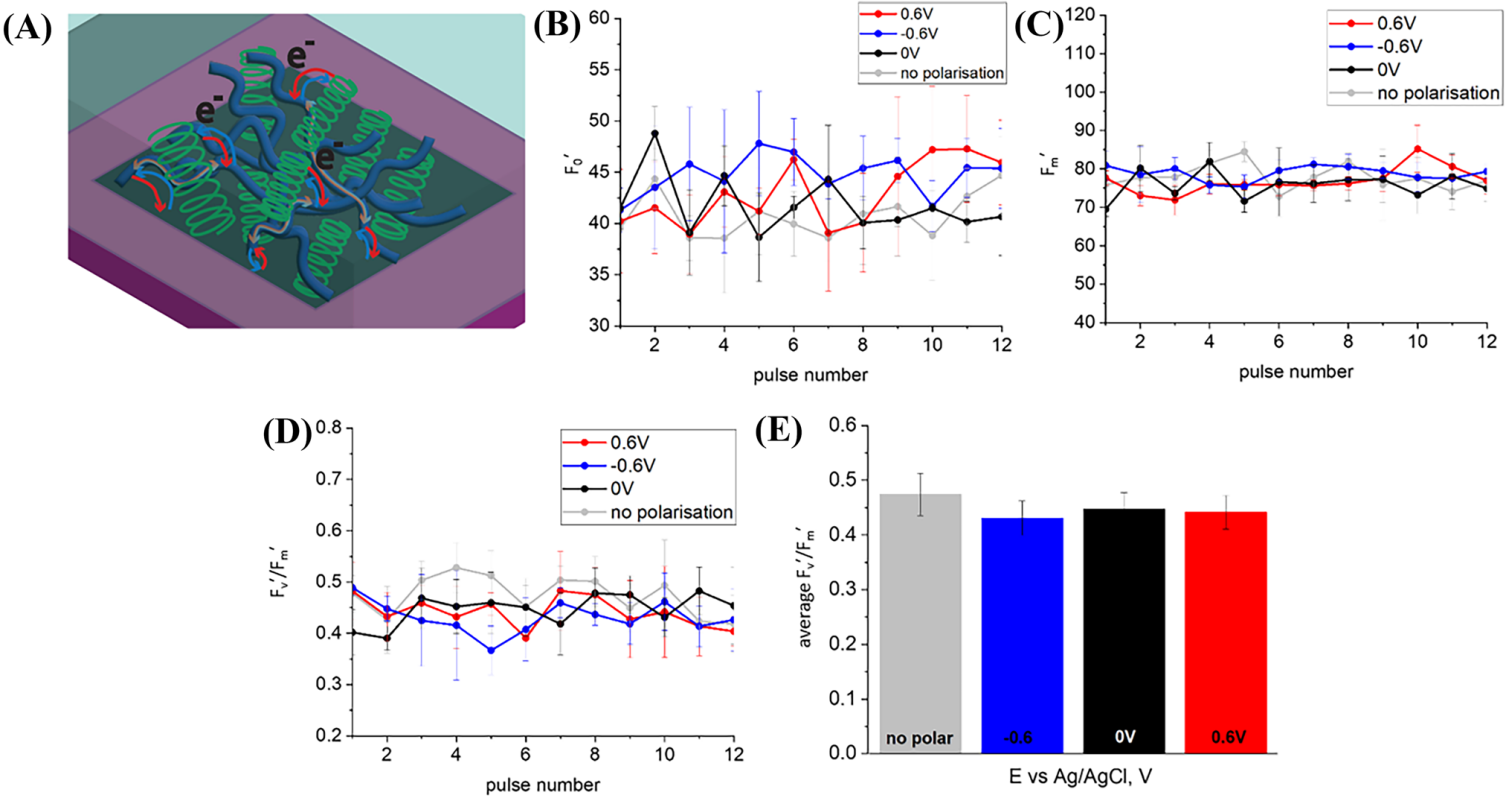
**A** Scheme of charge transfer associated with anodic (red arrow) and cathodic (blue arrow) currents in biophotoelectrode containing cyanobacteria embedded in PEDOT:PSS. **B** Data on steady-state fluorescence F_0_′ derived from PAM curves. **C** Data on the maximum chlorophyll fluorescence of light-adapted biophotoelectrodes. **D** The effective quantum yield of photoconversion calculated as (F_m_′ − F_0_′)/F_m_′. **E** Average effective quantum yield of photoconversion

Another approach to enhance charge transport from live cyanobacteria to the current collecting material is utilizing conductive polymers like PEDOT:PSS (Kayser and Lipomi 2019; Tseghai et al. 2020)( Fig. 9A) resulting in an increase of current generation at both positive and negative polarization (Fig. S2). Intensity of chlorophyll fluorescence, both minimal (Fig. 9B) and maximal (Fig. 9C) remains constant throughout measurement time and there is no or very little variation between different polarization conditions. Values of photochemical quantum yield are around 0.5, and no statistically significant differences (ANOVA, significance level 5%) between different potential biases were observed. However, comparison of different polarization conditions with OCP demonstrates statistically significant differences, with p-values of 0.02 and 0.003 for + 0.6 V and − 0.6 V, respectively.

Data on the maximum photoconversion yield F_v_/F_m_ and F_v_′/F_m_′ average for all tested systems and potentials are presented in Table 2, with only average values provided. As the PAM measurements were conducted under polarization controlled by the potentiostat, each PAM curve was recorded alongside the chronoamperometry curve (Fig. S2). Steady-state current data for each system is also included in Table 2.

**Table 2:** Comparison of photosynthetic performance in a BPV device with cyanobacteria immobilized in mediator-free agar, agar containing [Fe(CN)_6_]^4−^, agar containing [Fe(CN)_6_]^4−^in an electrolyte containing [Fe(CN)_6_]^4−^, and PEDOT:PSS.

	Agar				Agar + [Fe(CN)_6_]^4−^				Agar + [Fe(CN)_6_]^4−^ in PBS + [Fe(CN)_6_]^4−^				PEDOT:PSS		
E, V	no	−0.6	0	0.6	no	−0.6	0	0.6	no	−0.6	0	0.6	−0.6	0	0.6
I, μA	0	−1.2	0.09	24	0	−1.2	−0.08	54	0	−3.5	0.21	58	−28	−0.09	178
F_v_/F_m_	0.42	0.29	0.29	0.37	0.40	0.49	0.45	0.43	0.40	0.49	0.45	0.43	0.45	0.45	0.44
average F_v_′﻿/F_m_′	0.29	0.28	0.23	0.20	0.44	0.48	0.45	0.46	0.44	0.49	0.46	0.46	0.47	0.43	0.44
averageF_v_′﻿/F_m_′﻿ :F_v_/F_m_	0.69	0.96	0.79	0.54	1.10	0.98	1.00	1.07	1.10	1.00	1.02	1.07	1.04	0.96	1.00

Before the illumination period, cyanobacteria exhibited a maximum quantum yield of 0.4–0.5, indicating that only 50% of the light absorbed by the cyanobacteria could be utilized for photochemistry. This efficiency is lower than what has been recorded for algae biofilms (Herlory et al. 2007) but is compatible with data obtained for immobilized *Chlorella* (Ng et al. 2017).

As shown in the data above, polarization alone, in the absence of a durable electron transport pathway, can lead to a lower light utilization efficiency of *L. indica* (data for agar) compared to systems where EET is provided by charge shuttle ions ([Fe(CN)_6_]^4−^) or conductive polymer wires (PEDOT:PSS). Higher values of a quantum yield being a measure of the energetic efficiency of photoautotrophy indicate a superior optimization of the photochemical process. In this study, higher values for quantum yield in the light-acclimated state were measured for all systems where any component enhancing conductivity ([Fe(CN)_6_]^4−^ or PEDOT:PSS) was introduced. It facilitates reopening of reaction centers due to electron exchange and promotes higher light utilization efficiency.

The maximum quantum yield of *L. indica* cyanobacteria increased considerable (from 0.3 to 0.45–0.49) when cells were embedded in conductive matrix. Yet, both for *L. indica* contained in PEDOT:PSS as well as for *L. indica* encased in agar with [Fe(CN)_6_]^4−^, the average effective quantum yield is virtually the same as the maximum value measured for dark acclimated samples, whereas for the biophotoelectrode producing the lowest current (based on mediator-free agar), the quantum yield of light-adapted cells varied from 50% at + 0.6 V polarization to 100% at − 0.6 V polarization of the maximum photochemical yield.

### Light intensity dependence

**Fig. 10:**
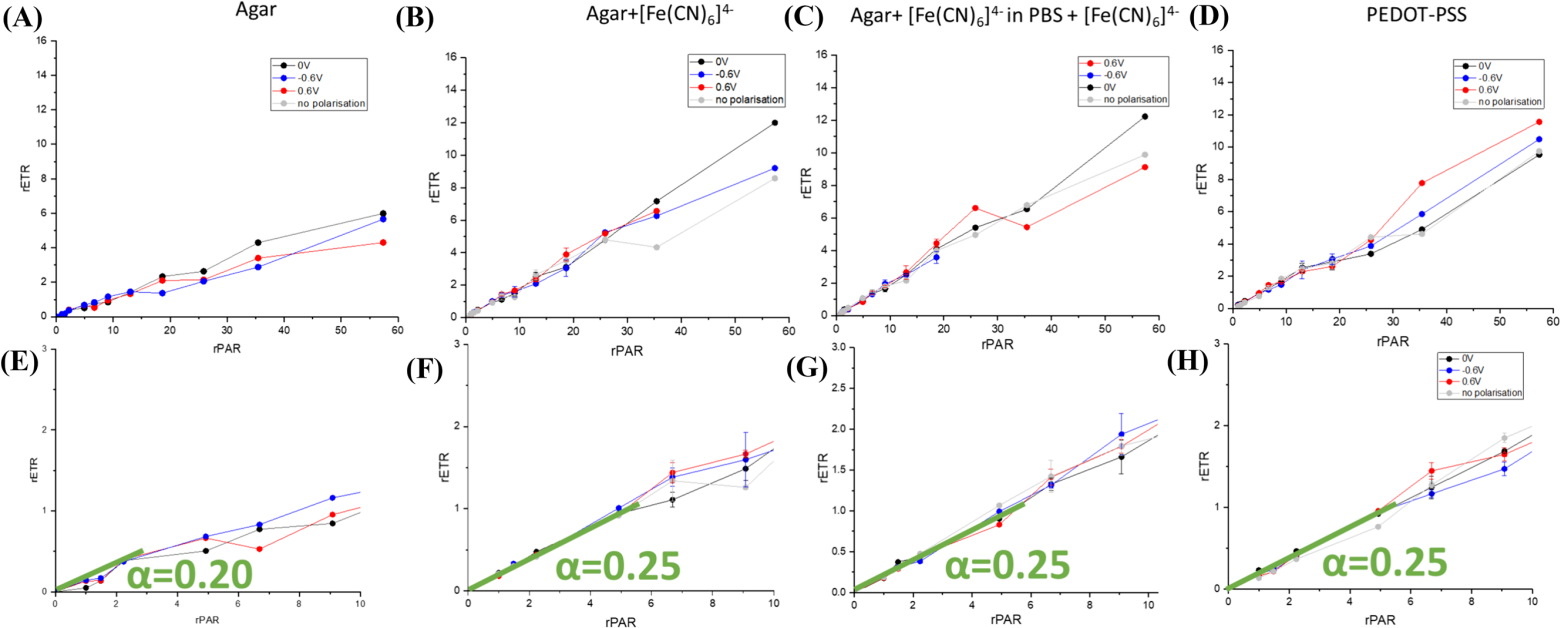
Light curves for cyanobacteria embedded in agar (**A**–**E**), agar+[Fe(CN)_6_]^4−^ (**B**–**F**), agar+[Fe(CN)_6_]^4−^ in electrolyte solution containing [Fe(CN)_6_]^4−^ (**C**–**G**), and PEDOT:PSS (**D**–**H**). **A**–**D** show the complete light curves, while **E**–**H** provide a magnified view of the light-limited conditions of the curves presented in **A**–**D** and α-value, the initial slope of the curve.

Other parameters such as the relative electron transport rate (rETR), rETR_max_ (the maximum rate of electron transport), and the light intensity of rETR saturation (E_k_), as well as α, the light utilization coefficient in light-limited conditions, determined as an initial slope in the ETR vs. irradiance (PAR) curve, were employed to assess the physiological state of the organism and were obtained using PAM chlorophyll fluorometry. Rapid light curves, which involve successive measurements on the same sample exposed to a stepwise increase in light intensity, are utilized to extract these data (Fig. 10). When cyanobacteria embedded in conductive PEDOT:PSS matrix electron transfer rate reached saturation (Fig. 10A) whereas this was not the case for the conductive systems (Fig. 10B–D). We expect that environmental stress lowers the threshold above which photosynthetic radiation becomes excessive. Hence, the addition of compounds capable of promoting charge transfer from cyanobacterial cells (via a positive voltage bias) and to cyanobacterial cells (via a negative voltage bias) is likely to promote excessive light protection mechanisms by enhancing charge transfer and preventing reduced intermediates accumulation.

The alpha value demonstrated in panels E to H of Fig. 10 is a critical parameter for characterizing the photosynthetic efficiency of photosynthetic organisms. It helps researchers to understand how efficiently these organisms use low-intensity light for photosynthesis. This value can be useful for optimizing conditions in biophotovoltaic and biophotoelectrochemical systems that rely on photosynthetic organisms for energy conversion. Surprisingly, the system which is expected to produce the lowest photocurrent (or not produce it at all), i.e., the one where the internal photosynthetic electron transport chain is not perturbed, demonstrated decreased efficiency at low light intensity conditions.

## Conclusions

This research contributes to the broader scientific understanding of cyanobacterial physiology and offers a foundation for developing sustainable applications harnessing the photosynthetic potential of these microorganisms. The integration of PAM technique with external polarization represents a novel approach in unraveling the intricate mechanisms governing cyanobacterial photosynthesis, fostering advancements in biotechnological and environmental applications. We observed that increasing the conductivity of the cyanobacteria embedding matrix enhances the efficiency of light utilization. Our investigations concerning the influence of the electrical polarization on the bioelectrochemical systems deployed in this study revealed small yet statistically significant differences in light utilization efficiency for PEDOT:PSS embedded *Limnospira*. Similar, additional research could be undertaken e.g. to determine the effects of higher bias magnitudes and longer durations of bias electric fields on light utilization efficiencies and the combined effects of such electrical bias variables with other environmental factors (including light intensity, wavelength, temperature, and nutrient availability). A thorough understanding of the dependence of light utilization efficiencies on externally applied polarizations (whether or not in combination with other parameters) has significant implications for augmenting the effectiveness of cyanobacterial-based technologies and long term operations. Given the large variety of cyanobacteria potentially suitable for bioelectrochemical applications such as biophotovoltaics, biosensors, and bioconversions and the present innovations in conductive materials (including semiconductors, redox mediators, and immobilizers), a comparative study of different cyanobacterial species and alternative charge collectors would be highly justifiable.

## Supplementary Information

Below is the link to the electronic supplementary material.

Supplementary file1 (DOCX 485 KB)

Supplementary file2 (TIF 968 KB)

Supplementary file3 (TIF 511 KB)

Supplementary file4 (TIF 422 KB)

## Data Availability

No datasets were generated or analysed during the current study.

## Abbreviations

Ag/AgCl: Silver silver-chloride
BDD: Boron-doped diamond
BPEC: Biophotoelectrochemical cell
Chl *a*: Chlorophyll *a*
DCMU: 3-(3,4-Dichlorophenyl)-1,1-dimethylurea
DLC: Diamond-like carbon
EET: Extracellular electron transport
ETR: Electron transport rate
F_0_: Minimal fluorescence with PSII open
F_0_′: Fluorescence under actinic light
F_m_: Fluorescence after dark adaptation
F_m_′: Fluorescence after light adaptation
F_v_: F_v_ = F_m_-F_0_
MWPECVD: Microwave plasma enhanced chemical vapor deposition
NPQ: Non-photochemical quenching
OCP: Open-circuit potential
Φ: Effective quantum yield
PAM: Pulse-amplitude-modulation
PAR: Photosynthetically active radiation
PBS: Phosphate buffer saline
PEDOT:PSS: Poly(3,4-ethylenedioxythiophene) polystyrene sulfonate
PQ: Photochemical quenching
PS II: Photosystem II
Q_A_: Primary quinone electron acceptor
rETR: Relative electron transport rate
TMB: Trimethylboron
