# Supplementary material for: Fluorescence and electron transfer of Limnospira indica functionalized biophotoelectrodes
Source: Photosynth Res. 2024 Aug 21;162(1):29–45. doi: 10.1007/s11120-024-01114-5 (PMC11413049; doi:10.1007/s11120-024-01114-5)
Supplement: Supplementary file 1 — Supplementary file1 (DOCX 485 KB) [file 11120_2024_1114_MOESM1_ESM.docx]

**Supporting Information**

**Pulse-Amplitude-Modulation Fluorescence of Limnospira indica functionalized boron doped diamond biophotoelectrodes under electrochemical bias**

Nikolay Ryzhkov^1*^, Nora Colson^1,2,3^, Essraa Ahmed^2,3^, Paulius Pobedinskas^2,3^, Ken Haenen^2,3^,

Paul J. Janssen^4^, Artur Braun^1*^

^1^ Empa. Swiss Federal Laboratories for Materials Science and Technology, Laboratory for High Performance Ceramics, CH – 8600 Dübendorf, Switzerland

^2^ Institute for Materials Research (IMO), Hasselt University, Wetenschapspark 1, B-3590 Diepenbeek, Belgium

^3^ IMOMEC, IMEC vzw, Wetenschapspark 1, B-3590 Diepenbeek, Belgium

^4^ Institute for Nuclear Medical Applications, Belgian Nuclear Research Centre, B-2400 Mol, Bel-gium

Corresponding authors: E-Mail: [artur.braun@alumni.ethz.ch](mailto:artur.braun@alumni.ethz.ch)

nrzhkv@gmail.com**
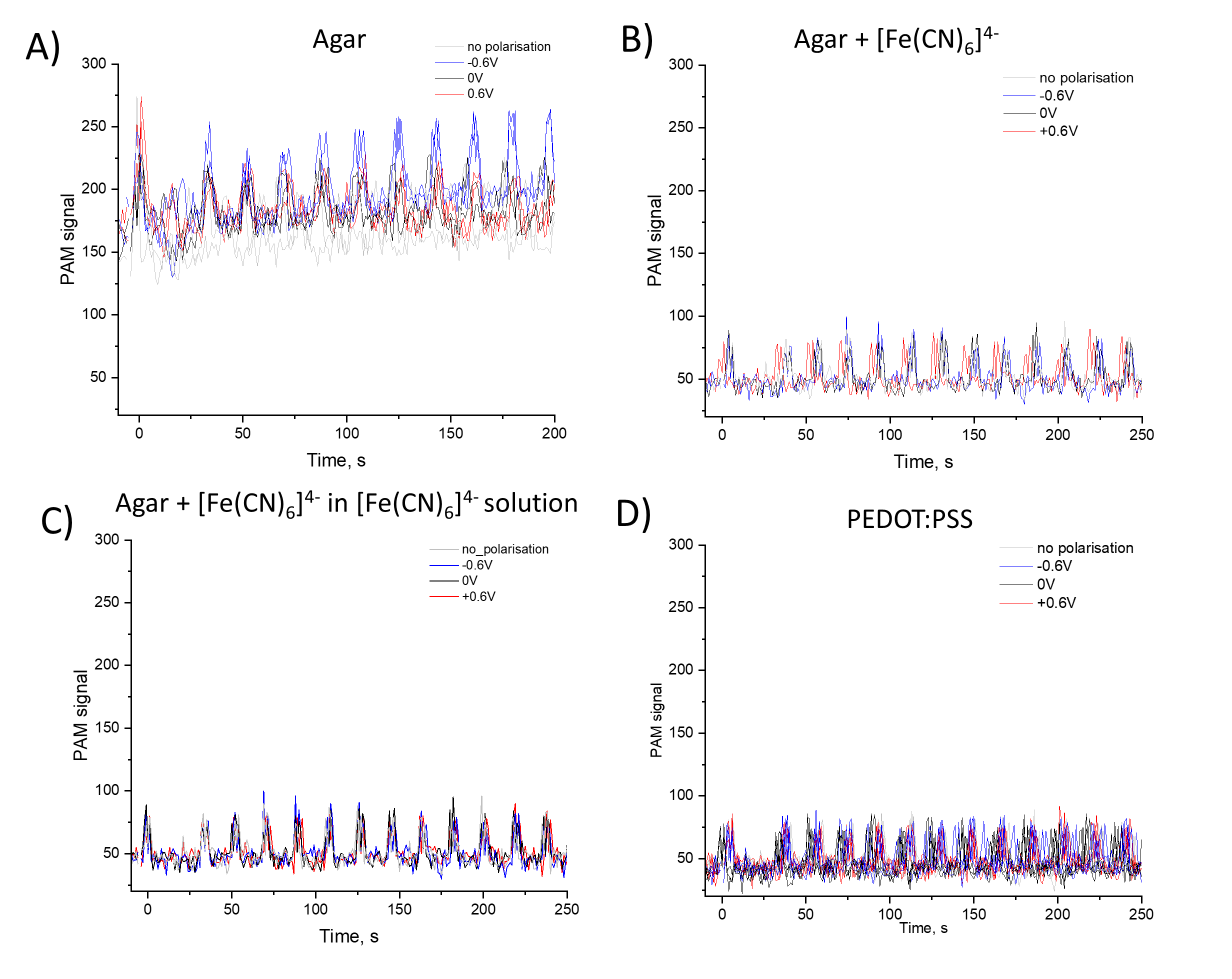
**

Figure S1. **PAM curves of cyanobacteria embedded in A) agar hydrogel, B) agar hydrogel containing [Fe(CN)_6_]^4-^, C) agar hydrogel containing [Fe(CN)_6_]^4-^ in [Fe(CN)_6_]^4-^ solution, D) PEDOT:PSS on top of a BDD working electrode polarized at 0V, +0.6V, and -0.6V vs. Ag/AgCl reference electrode in a 3-electrode cell.**


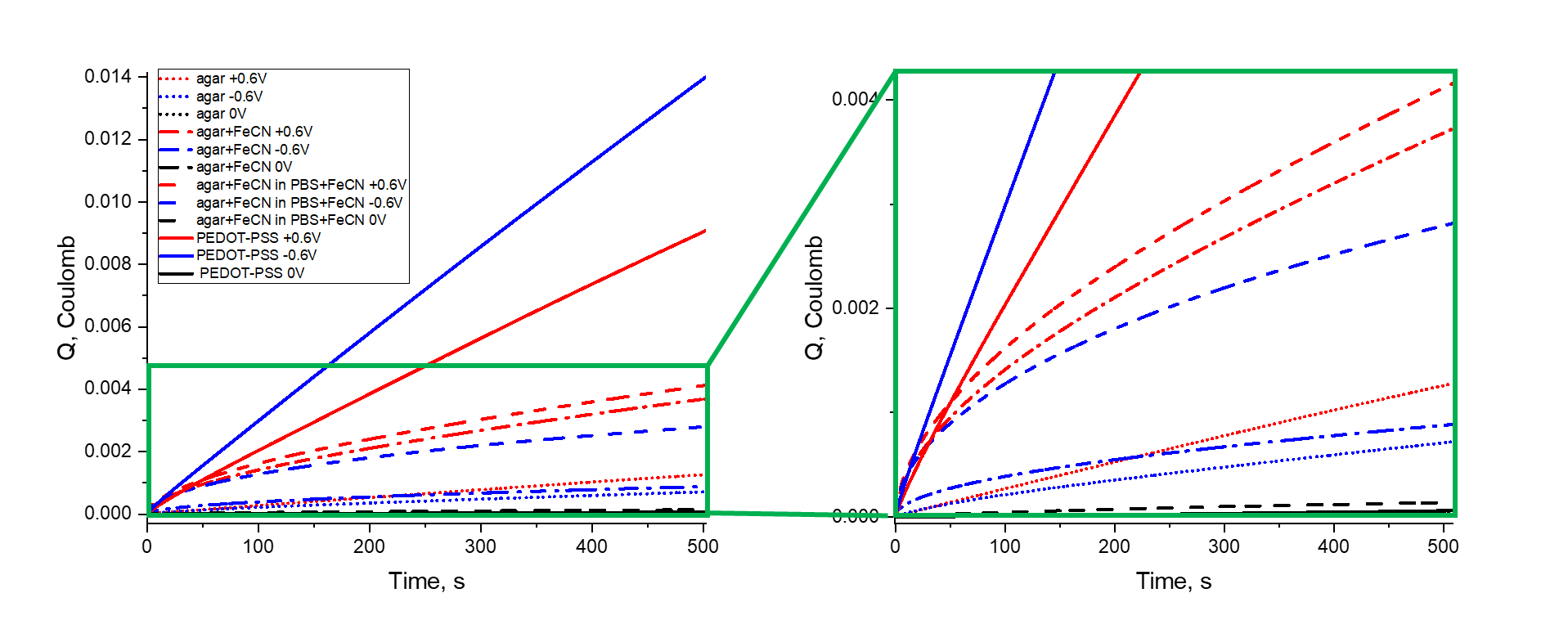


Figure S2. Currents measured for +0.6, 0 and -0.6V for cyanobacteria embedded in mediator-free agar, agar containing [Fe(CN)_6_]^4-^ and PEDOT:PSS in PBS solution and cyanobacteria embedded in agar containing [Fe(CN)_6_]^4-^in PBS solution containng [Fe(CN)_6_]^4-^ during PAM measurement.

**
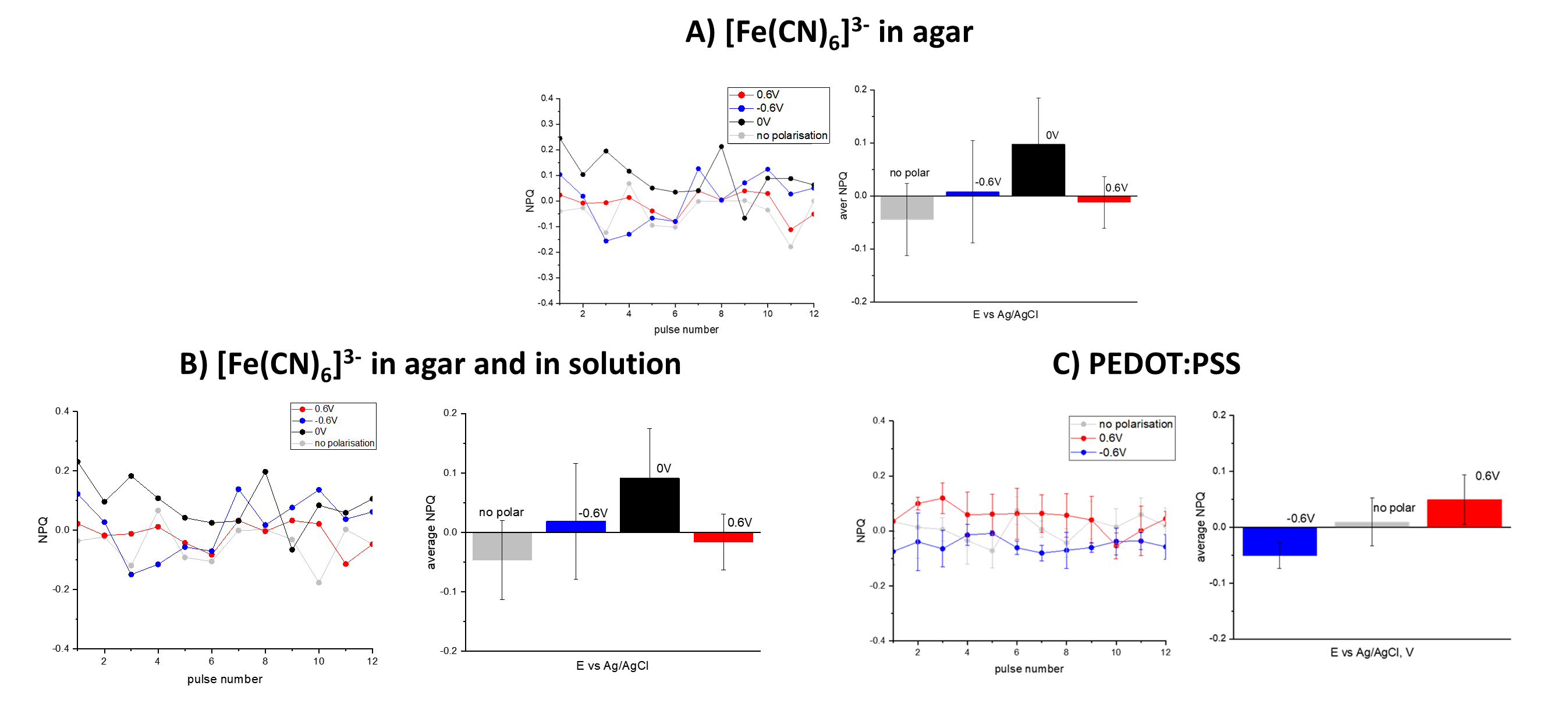
**Figure S3. NPQ values for cyanobacteria embedded in A) [Fe(CN)_6_]^4-^ containing agar in mediator free solution, B) [Fe(CN)_6_]^4-^ containing agar in [Fe(CN)_6_]^4-^ mediator containing solution, C) PEDOT:PSS.
