# Supplementary figures and images for: Fluorescence and electron transfer of Limnospira indica functionalized biophotoelectrodes
Source: Photosynth Res. 2024 Aug 21;162(1):29–45. doi: 10.1007/s11120-024-01114-5 (PMC11413049; doi:10.1007/s11120-024-01114-5)

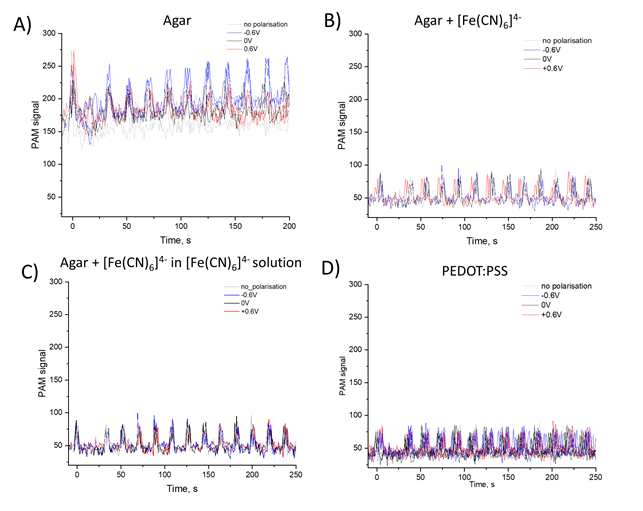

Supplement: Supplementary file 2 — Supplementary file2 (TIF 968 KB) [file 11120_2024_1114_MOESM2_ESM.tif]

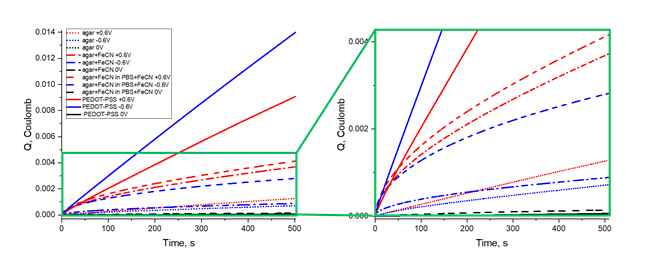

Supplement: Supplementary file 3 — Supplementary file3 (TIF 511 KB) [file 11120_2024_1114_MOESM3_ESM.tif]

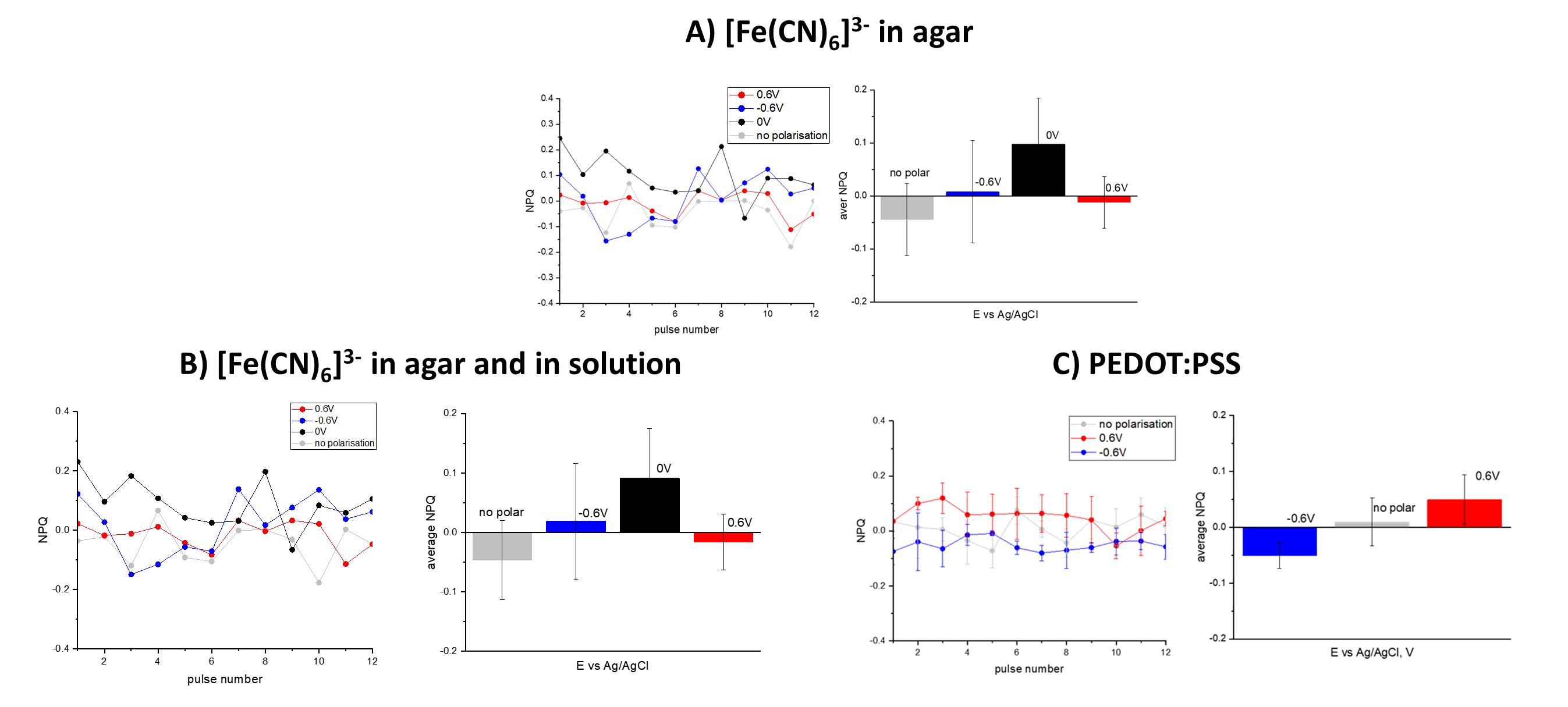

Supplement: Supplementary file 4 — Supplementary file4 (TIF 422 KB) [file 11120_2024_1114_MOESM4_ESM.tif]
